# Impact of Traumatic Childbirth and Birth‐Related Posttraumatic Stress Disorder on Breastfeeding Outcomes: A Systematic Review of Longitudinal and Cohort Studies

**DOI:** 10.1111/birt.70005

**Published:** 2025-10-06

**Authors:** Ester Cavallé‐Abasolo, Pelin Dikmen‐Yildiz, Irene Gómez‐Gómez, Lara Barros‐Martins, Emma Motrico

**Affiliations:** ^1^ Department of Psychology University Loyola Andalucía Sevilla Spain; ^2^ Department of Psychology Kirklareli University Kirklareli Turkey; ^3^ Department of Social Psychology School of Psychology, University of Seville Seville Spain; ^4^ Department of Developmental and Educational Psychology, School of Psychology University of Seville Seville Spain

**Keywords:** breastfeeding, cohort, longitudinal studies, posttraumatic stress disorder, systematic review, traumatic childbirth

## Abstract

**Background:**

Breastfeeding is the most recommended form of infant nutrition during the first months of life. Mother's perception of childbirth as traumatic, or birth‐related diagnosis of Posttraumatic Stress Disorder (PTSD) or symptoms (PTSS), may negatively affect breastfeeding outcomes, but there is not enough evidence about its influence. The aim of this study was to examine and summarize the available literature on the impact of traumatic childbirth and/or PTSD/PTSS related to childbirth on breastfeeding outcomes.

**Methods:**

Following PRISMA guidelines (PROSPERO: CRD42023407019), a systematic review of prospective longitudinal and cohort studies was conducted, involving searches across PubMed, PsycINFO, Scopus, Web of Science, and PsycARTICLES. The PICOS model guided inclusion criteria, and the Newcastle‐Ottawa Quality Assessment Scale (NOS) was used to assess study quality.

**Results:**

From the 1471 identified records, eight studies (involving 3091 participants) met our inclusion criteria and demonstrated an overall low risk of bias, according to NOS. Results consistently revealed a negative association between traumatic childbirth and/or birth‐related PTSS/PTSD and breastfeeding outcomes, including initiation, duration, self‐efficacy, and exclusive breastfeeding in the baby's early months.

**Discussion:**

A traumatic birth can have lasting effects on both maternal mental health and breastfeeding outcomes.

**Limitations:**

Potential omission of relevant studies despite searches across five databases and the absence of a calculated size effect, preventing the determination of the strength of the studied variables' relationship. Predominant focus on European studies questions the generalizability of the results.

**Conclusion:**

Mothers suffering from traumatic childbirth and/or childbirth‐related PTSS or PTSD have an increased risk of poorer breastfeeding outcomes.

**Registration and Protocol:**

The systematic review protocol was registered in PROSPERO. The registration number is: CRD42023407019.

## Graphical abstract

Traumatic childbirth effects on breastfeeding outcomes.

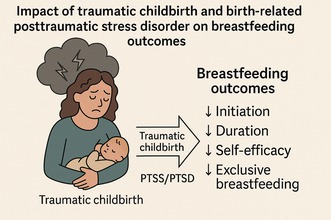

## 1 Background

Breastfeeding and human milk provide short‐ and long‐term medical and neurodevelopmental advantages to both children (e.g., lower risk of mortality and suffering many acute and chronic disorders, such as obesity) [1] and mothers (e.g., reduction of anxiety and negative mood and stress) [2, 3]. Exclusive breastfeeding is recommended for approximately 6 months after birth [1]. Moreover, the American Academy of Pediatrics (AAP) also advocates for continued breastfeeding for 2 years or beyond if the mother and the child would like to [1].

However, rates of breastfeeding differ according to sociodemographic and cultural factors. Low and middle‐income countries have higher rates of breastfeeding, while rates in high‐income countries seem to be lower [4]. Demographic and psychosocial factors also play an important role in decision‐making on whether to breastfeed or not, and when to cease breastfeeding (e.g., age and level of education of the mother [1] and support from a partner [5]). While sociodemographic and some psychosocial factors associated with breastfeeding (e.g., personality traits of the mother) are unchangeable, other psychological factors, such as self‐efficacy and confidence, are modifiable and of vital importance to encourage breastfeeding [6]. Moreover, the mother's perception of her childbirth experience plays an essential role in breastfeeding self‐efficacy, with women who perceive birth as a positive experience being the ones with higher breastfeeding self‐efficacy [7].

Traumatic childbirth is defined as “a woman's experience of interactions and/or events directly related to childbirth that caused overwhelming distressing emotions and reactions, leading to short and/or long‐term negative impacts on a woman's health and wellbeing” [8]. Women often feel vulnerable, disabled, and powerless, and have a sense of loss of control and panic [9]. Some studies suggest that the prevalence of mothers suffering from traumatic childbirth ranges from 20% to 30% [10], while others state that nearly 44% of mothers have a traumatic childbirth experience [11]. In some cases, traumatic childbirth leads to the development of Posttraumatic Stress Disorder (PTSD) symptoms, with a low proportion of women meeting all diagnostic criteria for PTSD [12]. In a meta‐analysis, the prevalence of PTSD after childbirth was 4% for community samples and 18.5% for women in high‐risk groups, with the latter including women with complications of pregnancy or birth [13].

The diagnostic criteria for PTSD after childbirth include (A) exposure to death, serious injury, or sexual violence, either experiencing it directly or witnessing it in the fetus or baby; (B) re‐experiencing the traumatic childbirth (e.g., flashbacks and intruding images); (C) avoidance of the stimuli associated with the trauma (e.g., photos or other pregnant women); (D) negative alterations in mood and cognitions (e.g., guilt and shame); (E) alterations in arousal and reactivity (e.g., easily being scared or angry); and (F) symptoms persist for at least 1 month and lead to clinically relevant distress or functional impairment [8, 14].

Although neither DSM‐IV nor DSM‐V specifically recognizes childbirth as an extreme traumatic stressor, criterion A can potentially lead childbirth to be categorized as a traumatic event. An instance is when the mother perceives a threat to her own and/or her infant's physical integrity [12].

There are several risk factors associated with the development of PTSS/PTSD after childbirth. Obstetric factors related to antepartum include a complicated course of pregnancy (e.g., preterm birth); peripartum variables include a complicated course of delivery (e.g., emergency C‐section, vacuum extraction, or postpartum hemorrhage); and postpartum variables include neonatal complications (e.g., NICU admission) [15, 16, 17]. Psychological risk factors include fear of childbirth, depression during pregnancy, psychiatric history, and previous psychological trauma, as well as stress and poor coping skills [15]. Finally, social factors such as psychosocial vulnerability (e.g., unwanted pregnancy, stressful relationship, work or health circumstances) and lack of practical and emotional support both in the perinatal and later in the postpartum period contribute to a higher likelihood of developing PTSS/PTSD after childbirth [15, 17]. Risk factors associated include those emerging before pregnancy (e.g., mother's age, antenatal education, and previous trauma), during the antenatal period (e.g., obstetric complications and unplanned pregnancy), during labor and birth (e.g., neonatal complications and perception of pain in labor), and in the postnatal period (e.g., low social support and insomnia) [18].

Furthermore, suffering from a traumatic childbirth and/or PTSS/PTSD after childbirth could have an impact on breastfeeding. Some women may be too overwhelmed by traumatic labor and delivery, which may be a barrier to initiating or continuing breastfeeding [19]. However, a qualitative study including women diagnosed with postpartum PTSD revealed that traumatic childbirth could lead to two extremely different breastfeeding experiences, one facilitating it and the other impeding it [20]. One of the factors that triggers maternal experiences of breastfeeding after a traumatic childbirth is intrusive flashbacks of the traumatic childbirth. These flashbacks have detrimental effects not only on the mothers but also on the neonate [20]. Consistent with DSM‐5 criteria, the baby may become a reminder of the traumatic delivery and lead the mother to re‐experience the event. This may result in the mother avoiding the baby, which in turn can lead to attachment problems [21].

On the other hand, breastfeeding can also be a useful way to overcome the negative effects of traumatic childbirth. Thus, the woman proves herself as a mother, bonds with her baby, and recovers emotionally after a difficult childbirth while breastfeeding [20]. The prevalence of PTSD after childbirth has been reported to be lower in mothers who breastfeed their babies within the first hour after birth [22], whereas those who breastfeed immediately after birth are at lower risk for developing PTSD [23].

Considering the above, there is a need to thoroughly understand the impact, if any, of traumatic childbirth and/or PTSS/PTSD on breastfeeding outcomes. To the best of our knowledge, no systematic review has investigated the association between traumatic childbirth and/or PTSS/PTSD and breastfeeding outcomes. There are two systematic reviews [24] and [25] on perinatal PTSD and child outcomes, but the focus was never or rarely placed on the impact of perinatal or birth‐related PTSD on breastfeeding. Therefore, the aim of this review was to summarize the literature available in longitudinal and cohort studies on the impact of traumatic childbirth and/or birth‐related postpartum PTSD or PTSD symptoms (PTSS) on breastfeeding outcomes.

## 2 Methods

This systematic review was conducted in accordance with the Preferred Reporting Items for Systematic reviews and Meta‐Analyses (PRISMA) guidelines [26]. The checklist of the PRISMA items can be found in Appendix A. The protocol of this study was previously registered with the International Prospective Register of Systematic Reviews database (PROSPERO) with registration number: *blinded_for_review*. No deviations were produced between the established protocol and the final review.

### 2.1 Search Strategy and Selection Criteria

Relevant studies were identified by searching the following five databases: PubMed, PsycINFO, Scopus, Web of Science, and PsycARTICLES. Search items were developed using keywords related to the perinatal period, PTSD, and breastfeeding. To develop a representative advanced search, MeSH terms were selected from the Medical Subject Headings (MeSH) thesaurus. The search was piloted in the PubMed database on May 8, 2023, and then adapted to the previously mentioned databases (see all searches in Appendix B). The search was conducted following the population/participants, intervention/key variable, comparison, outcome, and study design (PICOS) [27] model. Searches were conducted between May 8 and 11, 2023.

The inclusion criteria for the studies in this review were defined based on the PICOS model, as shown in Appendix C. Inclusion and exclusion criteria were selected to identify relevant studies assessing the impact of a traumatic childbirth and/or birth‐related PTSS/PTSD on breastfeeding outcomes, if any. The inclusion criteria were as follows: (a) participants were pregnant women or mothers to babies born alive and healthy; (b) perceived their childbirth as traumatic and/or suffered from birth‐related PTSD or PTSS; (c) study outcomes included breastfeeding outcomes; (d) prospective longitudinal or cohort design studies were selected to separate cause and effect over time. Case–control and cross‐sectional studies were excluded because they generally involve more bias than cohort studies [28]. No limits were imposed on the publication date and language.

### 2.2 Study Selection Process and Data Extraction

A reviewer (ECA) conducted the search, followed by the identification and removal of duplicates. ECA performed title and abstract screening under the supervision of EM. The remaining studies (*n* = 42) were full‐text reviewed according to inclusion and exclusion criteria by two groups of reviewers (ECA and LBM) and (ECA and IGG). In case of disagreement on the inclusion or exclusion of a study, the case was discussed to reach consensus. If necessary, a third reviewer (EM) was consulted to resolve any discrepancies. The reference lists of the included articles (*n* = 8) were screened to identify any additional relevant studies. Data extraction of the included studies was conducted by ECA and supervised by EM.

### 2.3 Quality Appraisal

The Newcastle‐Ottawa Quality Assessment (NOS) [29] was used to assess the quality of studies. NOS consists of eight items grouped into three categories. Each item assesses the characteristics of observational studies, including several answer options. Among the answer options, at least one is awarded a star. A star next to an answer indicates a low risk of bias. The final score is calculated by the sum of all stars awarded. Therefore, when a study is awarded a high number of stars, it means that it has good quality.

The maximum score on the NOS is 9 (highest quality), and total NOS scores are categorized into three groups: very high risk of bias (0–3 points), high risk of bias (4–6 points), and low risk of bias (7–9 points) [30]. The quality of each study was assessed based on the NOS scale by two independent reviewers (PDY and ECA). In case of disagreement, a third reviewer (EM) was consulted. The mean study quality score of the studies was 8.25 of 9 on the NOS, representing the high methodological quality of the studies included (see Appendix C).

## 3 Results

### 3.1 Study Selection

**FIGURE 1:**
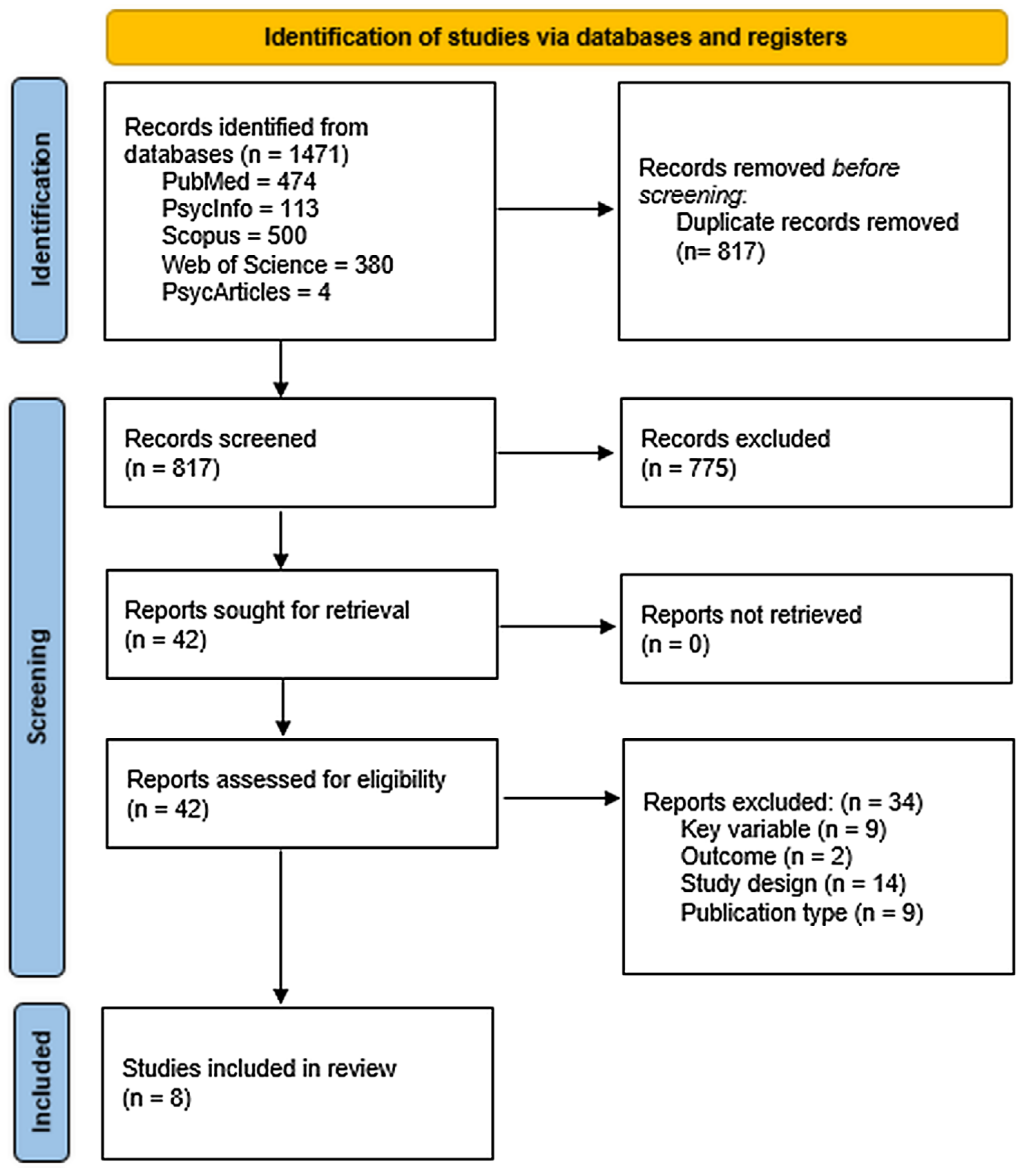
Study selection flow diagram. [Colour figure can be viewed at wileyonlinelibrary.com]

A total of 1471 records were obtained through the database search. See Figure 1 to review the screening process. A total of 34 studies were excluded at full text (see Appendix D), whereas eight studies were found to fulfill our inclusion criteria.

### 3.2 Study Characteristics

The characteristics of each study are summarized in Table 1. The eight studies were conducted in Greece [31], Norway [32], Israel [33], Italy [34], Brazil [35], Germany [36], Croatia [37] and Turkey [38] and were prospective longitudinal [31, 32, 34, 35, 36, 38], or cohort [33, 37] studies. The duration of follow‐up in longitudinal studies varied from 4 days [31] to 96 weeks [32]. The mean age of participants in the eight studies was 28.74 years. Sample sizes varied across studies, ranging from 95 [31] to 1480 participants [32] (mean *n* = 386.38). In total, this systematic review evaluated 3091 participants. Two studies [35, 37], with an experimental mortality of 35.1% and 30.1%, respectively, were included in the review.

**TABLE 1:** Characteristics of the included studies in the systematic review.

First author (year) region (country)	Design study (type)	Length of follow‐up	Target population (age)	Inclusion criteria	Exclusion criteria	Sample size at baseline	Assessment of traumatic childbirth and/or PTSD	Assessment of breastfeeding	Sample size at follow‐Up	Quality (NOS score)
Dimitraki (2016) Alexandroupolis (Greece)	Prospective longitudinal	4 days	Pregnant women (mean age 29, 2 years old)	Single pregnancy, no smoker, no other pathologies, delivered after 38th week, want to feed the baby with maternal milk, attend training course in breastfeeding during pregnancy	None	*n* = 100	Perinatal PTSD Questionnaire	(1) Hour of first breastfeeding and breast‐filling, (2) milk volume of the first 24 h and on the 4th day, and (3) newborns' meal frequency on the 4th day after delivery	*n* = 95	Low risk of bias
Garthus‐Niegel (2018) Oslo (Norway)	Prospective longitudinal	2 years	Pregnant women (mean age 31, 7 years old)	None	None	*n* = 3,752	IES	Questionnaire about (1) initiation; (2) exclusive breastfeeding at 2 years postpartum; (3) continued breastfeeding at 2 years postpartum	*n* = 1,480	Low risk of bias
Halperin (2015) Northern Israel (Israel)	Prospective cohort	6–8 weeks	Pregnant women (aged 18–41 years old)	Women, after their first or second birth, spoke Hebrew and gave birth at 34–42 weeks gestational age to a living single child	None	*n* = 230	PSS‐SR and self‐report questionnaire of assessment of childbirth as traumatic (yes/no)	Self‐report questionnaire asking about breastfeeding (yes/no).	*n* = 171	Low risk of bias
Lindau (2014) Rome (Italy)	Prospective longitudinal	24 weeks	Mothers (mean age 31, 3 years old)	Being able to be contacted by telephone, women's babies were not hospitalized immediately after birth	None	*n* = 605	Standardized questionnaire administered at hospital by trained interviewers of self‐perception of the birth experience as traumatic (yes/no)	Questionnaires administered by phone every 2 weeks for 6 months postpartum about breastfeeding practice, duration, and exclusiveness based on WHO definitions	*n* = 542	Low risk of bias
Machado (2014) Viçosa (Brazil)	Prospective longitudinal	16 weeks	Mothers aged 13–44 years old who gave birth in the city of Viçosa	Mothers of infants born in the period from October 2011 to April 2012	Hospitalization of the infant in the NICU*, syndromes or malformations, twin pregnancy	*n* = 259	Questionnaire to the mother about self‐report of birth as traumatic	Questionnaire to mother asking about exclusive breastfeeding (yes/no) at 1, 2, and 4 months after birth	*n* = 168	Low risk of bias
Martini (2022) Dresden (Germany)	Prospective longitudinal	64 weeks	Pregnant women (aged 18–40 years old)	Less than 12 weeks of gestation, more than 18 and younger than 40, single pregnancy, maximum three spontaneous abortions, no severe physical disease, no substance abuse in the last 6 months, no psychiatric illness, sufficient mastery of German	No	*n* = 306	Section N adapted to birth experience of the CIDI	Baby‐DIPS	*n* = 274	Low risk of bias
Srkalović imširagić (2016) Zagreb (Croatia)	Prospective cohort	6–9 weeks	Mothers (aged 15–45 years old)	Having delivered in the study hospital during the data collection period, having permanent domicile address, speaking Croatian, and not undergoing psychiatric treatment	No	*n* = 372	IES‐R	Self‐reported questionnaire on exclusive breastfeeding status	*n* = 259	Low risk of bias
Türkmen (2020) all regions (Turkey)	Prospective longitudinal	24 weeks	Pregnant women (aged 18–40 years old)	Having term pregnancy, planning a vaginal delivery, and having a healthy singleton pregnancy	No	*n* = 130	TCPS and PTSD‐Short Scale	BSES	*n* = 102	Low risk of bias

Abbreviations: Baby‐DIPS, Composite International Diagnostic Interview for Women; BSES, Breastfeeding Self‐Efficacy Scale; CIDI: Composite International Diagnostic Interview; IES, Impact of Events Scale; IES‐R, Impact of Events Scale‐Revised; PSS‐SR, PTSD Symptom Scale; PTSD, Posttraumatic stress disorder; PTSD‐Short Scale, Post‐Traumatic Stress Disorder‐Short Scale; TCPS, Traumatic Childbirth Perception Scale.

The variables of interest were assessed using different measures. Most studies used standardized, validated scales to assess birth‐related PTSD, including the Turkish version of the PTSD‐Short Scale [39]; the Perinatal PTSD Questionnaire [40]; and the Impact of Events Scale (IES) [41]. A study [36] used section N of the Composite International Diagnostic Interview (CIDI) by adapting its items to the birth experience [42]. Two studies used the Impact of Events Scale–Revised (IES‐R) [43] to assess PTSS [37]. Traumatic childbirth was assessed via a questionnaire about self‐perception of the birth experience as traumatic or not (yes/no), either self‐administered [33, 35] or administered by trained reviewers [34]. A study [38] was based on the Traumatic Childbirth Perception Scale (TCPS) [44].

To assess breastfeeding outcomes, a study [31] used physiological measures (milk volume, time at first breastfeeding, and breast filling and feeding frequency of the baby). The remaining studies (*n* = 7) used a variety of self‐report questionnaires, such as the Structured Diagnostic Interview (Baby‐DIPS) [45], the Breastfeeding Self‐Efficacy Scale (BSES) [38, 46], or questionnaires created ad hoc based on WHO definitions [32, 34, 35].

## 4 Main Results

Table 2 summarizes the main results of the studies included. The eight studies consistently demonstrated an association between traumatic childbirth and/or birth‐related PTSS/PTSD and the outcome of breastfeeding. However, in three studies [34, 36, 37], this association only reached significance when these disorders were concomitant with other psychological distress conditions [34, 36] or after adjustment for other sociodemographic and obstetric variables [37].

**TABLE 2:** Main results of the included studies in the systematic review.

Authors (year)	Traumatic childbirth and/or PTSD/PTSS measured	Breastfeeding outcome	Statistical technique/s	Association between traumatic childbirth and/or PTSD and breastfeeding (yes/No)	Results
Dimitraki et al. (2016)	PTSD	Lactogenesis and lactation outcomes (primary outcome), measured during the first 4 days after birth	(1) r Pearson correlation coefficient and (2) model of multiple regressions	Yes	Levels of negative feelings and posttraumatic stress mothers experienced because of the labor were strongly correlated with lactogenesis markers (milk volume on the 4th day, meal frequency, breast filling sense, first feeding after delivery)
Garthus‐Niegel et al. (2018)	PTSD	Breastfeeding initiation, exclusive breastfeeding during the first 6 months, and continuation up to 1 and 2 years (primary outcomes)	(1) Bivariate Pearson (*r*) and phi coefficient (*r*_*φ*_) correlations to estimate PP‐PTSD and other maternal and child factors, (2) stepwise logistic regression analyses with forward selection, and (3) bivariate associations	Yes	Women with PTSD due to childbirth were less likely to initiate breastfeeding. Continuation of breastfeeding up to 1 year was negatively associated with PTSD due to childbirth, but only at bivariate level. Exclusive breastfeeding during the first 6 months and continuation of breastfeeding up to 2 years were not significantly associated with PTSD due to childbirth
Halperin et al. (2015)	Traumatic childbirth and PTSS	Breastfeeding rates at 6–8 weeks postpartum (secondary outcome)	(1) *X* ^2^ tests, (2) *t*‐tests, and (3) hierarchical linear regression in four steps	Yes	Significantly more women with PTSD symptoms 6–8 weeks after childbirth were not breastfeeding
Lindau et al. (2014)	Traumatic childbirth	EBF for at least 4 months (primary outcome)	(1) Unconditional logistic regression analysis to identify factors associated with exclusive breastfeeding for at least 4 months, and (2) multivariate analysis with variables with a *p* ≤ 0.05	Yes, but only significant when accompanied by other psychologically distressful conditions	Perception of birth as a traumatic event is associated with increased odds of stopping EBF before 4 months, although it did not reach statistical significance. However, women with the presence of two or more psychological distress conditions (depression episodes in a lifetime, insomnia, and experiencing birth as a traumatic event) versus none were three times more likely to stop exclusive breastfeeding before 4 months
Machado et al. (2014)	Traumatic childbirth	Early EBF abandonment (primary outcome), measured at 1, 2, and 4 months after birth	(1) Bivariate analyses using the Pearson's Chi‐square test, the linear trend test, or Fisher's test, (2) multivariate analysis using Poisson regression with robust variance adjustment, (3) stepwise backward selection procedure, and (4) survival analysis	Yes	Mothers who had a traumatic delivery had a significantly higher chance of EBF abandonment at 2 months. This was shown both in the bivariate analysis and the multivariate analysis
Martini et al. (2022)	PTSD	Duration of breastfeeding and feeding problems of the infant (primary outcome), measured at 2, 4, and 14 months after birth	Separated linear and logistic regression analyses for each outcome variable	Yes, but only significant when accompanied by depression of the mother	Infants of mothers with birth‐related traumatization and postpartum depression were at higher risk for feeding problems
Srkalović imširagić et al. (2016)	PTSS	EBF 6–9 weeks after delivery (primary outcome)	(1) Spearman's rank correlation, (2) univariate binary logistic regression analysis, (3) Multivariate, adjusted binary logistic regression analysis, and (4) Johnson–Neyman technique	Yes, but only significant at univariate level	PTSD symptoms reduce chances for EBF at 6–9 weeks after delivery at univariate level but not at multivariate level
Türkmen et al. (2020)	Traumatic childbirth and PTSD	Breastfeeding self‐efficacy after the fourth postpartum week (primary outcome), measured at 4 weeks and 3 months after birth	(1) Independent samples *t*‐test, (2) analysis of variance (ANOVA), (3) Pearson's correlation test, and (4) multiple linear regression analysis	Yes	Women with high traumatic childbirth perception levels had significantly lower breastfeeding self‐efficacy levels than those with moderate and low traumatic childbirth perception levels. There is a significant relationship between traumatic childbirth perception, PTSD, and breastfeeding self‐efficacy 4 weeks and 3 months after birth

## 5 Discussion

This is the first systematic review that demonstrates that traumatic childbirth and/or birth‐related PTSS/PTSD might have deleterious effects on breastfeeding outcomes. In other words, mothers perceiving their childbirth as traumatic or suffering birth‐related PTSS or PTSD are more likely to show poor lactogenesis markers during the first days postpartum, have lower rates of exclusive and non‐exclusive breastfeeding, breastfeeding initiation and duration, and lower breastfeeding self‐efficacy. However, there was a high heterogeneity in the results of the review, as many of the studies found that the association between variables was only significant when there was other concomitant psychological distress, or the models were adjusted for other covariates.

These findings arise from eight prospective longitudinal and cohort studies, including 3091 women from eight countries and three continents (Europe, South America, and Asia). The quality of studies was assessed using the NOS scale as low risk of bias, which demonstrated a high methodological quality of the studies.

### 5.1 Comparison With Previous Literature

Our results are in line with previous reviews of risk factors for PTSS/PTSD that also found that traumatic birth could negatively impact breastfeeding outcomes [18]. The involvement of physiological pathways could explain the negative relationship between traumatic childbirth and/or childbirth‐related PTSS/PTSD and poorer breastfeeding outcomes. A study included in this review [31] used physiological measures to assess breastfeeding. The findings of this study parallel those of previous studies [47], in which breast fullness appeared later in mothers with higher cortisol and glucose levels and prolonged labor. Also, milk volume on the fifth day postpartum was lower in women with higher cord glucose levels. The explanation for this could lie in the release of oxytocin induced by physical and mental stress, which impairs the milk ejection reflex of the mother and, when stress is prolonged, may prevent breast fullness in each feed [48]. Although stress during labor is normal and facilitates lactogenesis, experiencing high levels of stress has detrimental effects on lactogenesis [47, 49].

Furthermore, psychological pathways also have effects on breastfeeding outcomes. One of the most important symptoms of PTSD is avoidance of the stimuli related to the traumatic event [14]. In this situation, the mother rejects contact with the baby, as it is a constant reminder of the trauma [9, 50]. This psychological mechanism may explain the stronger relationship observed between avoidance symptoms and non‐initiation or non‐continuation of breastfeeding up to 1 year postpartum [32]. Moreover, some mothers perceived breastfeeding as another way to be violated, experienced intrusive flashbacks of the traumatic childbirth that impeded breastfeeding, and felt distanced and detached from their babies [20]. Furthermore, women with PTSS have more cognitive biases in recognizing emotional expressions and are less sensitive to the needs of the baby [51], which can, in turn, negatively affect breastfeeding.

One of the studies included in this systematic review showed an association between childbirth‐related PTSD and exclusive breastfeeding at 1 year postpartum, but no association at 6 months postpartum [32]. This finding could be explained by the tendency to recover spontaneously from PTSD reported in other studies included in this review [32, 35, 38].

Among the different breastfeeding outcomes that have been examined in this systematic review, breastfeeding self‐efficacy was only assessed in a study [38], finding a significant negative relationship between traumatic childbirth and breastfeeding self‐efficacy. In the literature, breastfeeding self‐efficacy is found to predict breastfeeding success at 1 and 2 months after childbirth [52]. Also, higher levels of breastfeeding self‐efficacy promote perseverance, self‐confidence, and the ability to go through the challenges of breastfeeding [53]. In addition, the efficacy of interventions that promote breastfeeding self‐efficacy has been demonstrated; mothers receiving these interventions are more likely to be breastfeeding at 1 and 2 months after childbirth [52]. Therefore, these interventions for improving breastfeeding self‐efficacy should be considered in cases where the mother suffered a traumatic childbirth and/or has PTSS that can undermine breastfeeding self‐efficacy.

Two studies in this review [42, 44] revealed a relationship between PTSD or traumatic childbirth and breastfeeding outcomes only when they were concurrent with postpartum depression or depression in the lifetime. These findings are consistent with a growing body of evidence that the comorbidity of childbirth‐related PTSD with depression is common in pregnancy and after birth [54]. Thus, seven out of nine of the mothers with postpartum PTSD also suffer from depression at 6 months postpartum [55]. Moreover, depression before and during pregnancy is a risk factor for the development of childbirth‐related PTSD [54, 56].

In the light of these findings, a traumatic childbirth can have long‐lasting effects on breastfeeding outcomes and mothers' mental health. Thus, support networks should be available for mothers to prevent childbirth‐related PTSS/PTSD and its negative impact on breastfeeding. Health care professionals should evaluate the relationship that mothers with PTSS have with their babies, as they may need intensive one‐to‐one support from a breastfeeding counselor. Also, governmental and nongovernmental breastfeeding support services, such as virtual breastfeeding support groups, should be a priority. Additionally, midwives, gynecologists, and mental health professionals should receive specific training in providing effective breastfeeding support. This way, they would be able to determine when a mother needs to be referred to other professionals who help her improve her breastfeeding experience. Moreover, early screening for postpartum PTSD should be performed, and preventive measures should be adopted to ensure that childbirths are more respectful of the needs of mothers. In the workplace, maternity leave should be extended to 6 months in order to facilitate the 6‐month exclusive breastfeeding period recommended by the AAP and the WHO [1], and health services should offer educational activities to promote breastfeeding.

## 6 Strengths and Limitations

This systematic review has some strengths and limitations that need to be mentioned. Firstly, although the search for the studies was conducted on five representative and well‐known databases, we still may have missed some potentially eligible studies that may not be included in those databases. Secondly, another limitation is that the size effect of the results has not been calculated, so we do not know the strength of the relationship between the variables studied. Moreover, all the studies included in this systematic review were conducted in Europe, except for two, which were conducted in Brazil (South America) and Israel (Asia). Therefore, the results may not be representative of mothers from all countries. Further studies are needed to ensure the external validity of results in other countries.

## 7 Conclusion

Mothers suffering from traumatic childbirth and/or childbirth‐related PTSS or PTSD have an increased risk of poorer breastfeeding outcomes, such as lower rates of initiation and duration of both exclusive and nonexclusive breastfeeding and lower breastfeeding self‐efficacies. Further research is needed to better understand the impact of traumatic childbirth and/or childbirth‐related PTSS/PTSD on breastfeeding outcomes. Specific healthcare policies are needed to prevent this disorder and promote breastfeeding.

## Conflicts of Interest

The authors declare no conflicts of interest.

## Data Availability

Data sharing is not applicable to this article as no new data were created or analyzed in this study.

## Appendix A

PRISMA checklist 2020.

**Table:**

Section and Topic	Item #	Checklist item	Reported on page #
TITLE			
Title	1	Identify the report as a systematic review.	1
ABSTRACT			
Abstract	2	Provide a structured summary, including, as applicable: background; objectives; study eligibility criteria; data sources; participants and interventions; study appraisal and synthesis methods; results; limitations; conclusions and implications of key findings; systematic review registration number.	3
INTRODUCTION			
Rationale	3	Describe the rationale for the review in the context of existing knowledge.	4‐11
Objectives	4	Provide an explicit statement of the objective(s) or question(s) the review addresses.	11
METHODS			
Eligibility criteria	5	Specify the inclusion and exclusion criteria for the review and how studies were grouped for the syntheses.	12
Information sources	6	Specify all databases, registers, websites, organisations, reference lists and other sources searched or consulted to identify studies. Specify the date when each source was last searched or consulted.	13
Search strategy	7	Present the full search strategies for all databases, registers and websites, including any filters and limits used.	14
Selection process	8	Specify the methods used to decide whether a study met the inclusion criteria of the review, including how many reviewers screened each record and each report retrieved, whether they worked independently, and if applicable, details of automation tools used in the process.	13
Data collection process	9	Specify the methods used to collect data from reports, including how many reviewers collected data from each report, whether they worked independently, any processes for obtaining or confirming data from study investigators, and if applicable, details of automation tools used in the process.	13
Data items	10a	List and define all outcomes for which data were sought. Specify whether all results that were compatible with each outcome domain in each study were sought (e.g., for all measures, time points, analyses), and if not, the methods used to decide which results to collect.	12
	10b	List and define all other variables for which data were sought (e.g., participant and intervention characteristics, funding sources). Describe any assumptions made about any missing or unclear information.	12
Study risk of bias assessment	11	Specify the methods used to assess risk of bias in the included studies, including details of the tool(s) used, how many reviewers assessed each study and whether they worked independently, and if applicable, details of automation tools used in the process.	31‐32
Effect measures	12	Specify for each outcome the effect measure(s) (e.g., risk ratio, mean difference) used in the synthesis or presentation of results.	N/A
Synthesis methods	13a	Describe the processes used to decide which studies were eligible for each synthesis (e.g., tabulating the study intervention characteristics and comparing against the planned groups for each synthesis ((item #5))).	13
	13b	Describe any methods required to prepare the data for presentation or synthesis, such as handling of missing summary statistics, or data conversions.	N/A
	13c	Describe any methods used to tabulate or visually display results of individual studies and syntheses.	N/A
	13d	Describe any methods used to synthesize results and provide a rationale for the choice(s). If meta‐analysis was performed, describe the model(s), method(s) to identify the presence and extent of statistical heterogeneity, and software package(s) used.	N/A
	13e	Describe any methods used to explore possible causes of heterogeneity among study results (e.g., subgroup analysis, meta‐regression).	N/A
	13f	Describe any sensitivity analyses conducted to assess robustness of the synthesized results.	N/A
Reporting bias assessment	14	Describe any methods used to assess risk of bias due to missing results in a synthesis (arising from reporting biases).	13
Certainty assessment	15	Describe any methods used to assess certainty (or confidence) in the body of evidence for an outcome.	N/A
RESULTS			
Study selection	16a	Describe the results of the search and selection process, from the number of records identified in the search to the number of studies included in the review, ideally using a flow diagram.	15‐16
	16b	Cite studies that might appear to meet the inclusion criteria, but which were excluded, and explain why they were excluded.	69‐71
Study characteristics	17	Cite each included study and present its characteristics.	17‐24
Risk of bias in studies	18	Present assessments of risk of bias for each included study.	33
Results of individual studies	19	For all outcomes, present, for each study: (a) summary statistics for each group (where appropriate) and (b) an effect estimate and its precision (e.g., confidence/credible interval), ideally using structured tables or plots.	25‐27
Results of syntheses	20a	For each synthesis, briefly summarise the characteristics and risk of bias among contributing studies.	31‐33
	20b	Present results of all statistical syntheses conducted. If meta‐analysis was done, present for each the summary estimate and its precision (e.g., confidence/credible interval) and measures of statistical heterogeneity. If comparing groups, describe the direction of the effect.	N/A
	20c	Present results of all investigations of possible causes of heterogeneity among study results.	N/A
	20d	Present results of all sensitivity analyses conducted to assess the robustness of the synthesized results.	N/A
Reporting biases	21	Present assessments of risk of bias due to missing results (arising from reporting biases) for each synthesis assessed.	N/A
Certainty of evidence	22	Present assessments of certainty (or confidence) in the body of evidence for each outcome assessed.	N/A
DISCUSSION			
Discussion	23a	Provide a general interpretation of the results in the context of other evidence.	34‐40
	23b	Discuss any limitations of the evidence included in the review.	40
	23c	Discuss any limitations of the review processes used.	40
	23d	Discuss implications of the results for practice, policy, and future research.	41
OTHER INFORMATION			
Registration and protocol	24a	Provide registration information for the review, including register name and registration number, or state that the review was not registered.	11
	24b	Indicate where the review protocol can be accessed, or state that a protocol was not prepared.	11, 42
	24c	Describe and explain any amendments to information provided at registration or in the protocol.	N/A
Support	25	Describe sources of financial or non‐financial support for the review, and the role of the funders or sponsors in the review.	42
Competing interests	26	Declare any competing interests of review authors.	42
Availability of data, code, and other materials	27	Report which of the following are publicly available and where they can be found: template data collection forms; data extracted from included studies; data used for all analyses; analytic code; any other materials used in the review.	N/A

## Appendix B

Search strategy in five databases.

Search strategy in PubMed.

**Table:**

((((((((((((((((((((((((postpartum[Title/Abstract]) OR postnatal[Title/Abstract]) OR puerperal[Title/Abstract]) OR perinatal[Title/Abstract]) OR prenatal[Title/Abstract]) OR antenatal[Title/Abstract]) OR intrapartum[Title/Abstract]) OR pregnancy[Title/Abstract]) OR “pregnancy”[MeSH Terms]) OR “pregnant women”[Title/Abstract]) OR “pregnant women”[MeSH Terms]) OR matern*[Title/Abstract]) OR “postpartum”[tw]) OR “post‐partum”[tw]) OR “post partum”[tw]) OR “puerperium”[tw]) OR “Perinatal care”[Mesh Terms]) OR “perinat*”[tw]) OR “postnat*”[tw]) OR “maternal”[tw]) OR “Prenatal Care”[Mesh Terms]) OR “prenatal”[tw]) OR “antenatal”[tw]) OR “Puerperal Disorders”[Mesh Terms]) AND (((((((((((((((((((((((((((((((((((((((((“Stress Disorders, Post‐Traumatic”[Mesh Terms]) OR “Stress Disorder, Posttraumatic”[Title/Abstract]) OR “Stress Disorder, Post Traumatic”[Title/Abstract]) OR “Post‐Traumatic Stress Disorder”[Title/Abstract]) OR “Posttraumatic Stress Disorder”[Title/Abstract]) OR “Post Traumatic Stress Disorder”[Title/Abstract]) OR “Acute Post‐Traumatic Stress Disorder”[Title/Abstract]) OR “Acute Post Traumatic Stress Disorder”[Title/Abstract]) OR ptsd[Title/Abstract]) OR “Neuroses, Post‐Traumatic”[Title/Abstract]) OR “Neuroses, Post Traumatic”[Title/Abstract]) OR “Post‐Traumatic Neuroses”[Title/Abstract]) OR “Posttraumatic Neuroses”[Title/Abstract]) OR “posttraumatic neuros*”[Title/Abstract]) OR “Post‐Traumatic stress symptoms”[Title/Abstract]) OR “Stress Disorders, Traumatic”[Mesh Terms]) OR “Stress Disorder, Traumatic”[Title/Abstract]) OR “Traumatic Stress Disorder*”[Title/Abstract]) OR “Traumatic Disorder”[Title/Abstract]) OR “traumatic stress”[Title/Abstract]) OR “stress disorder*”[Title/Abstract]) OR post‐traumatic[Title/Abstract]) OR “post traumatic”[Title/Abstract]) OR posttraumatic[Title/Abstract]) OR posttrauma[Title/Abstract]) OR traumatic[Title/Abstract]) OR trauma[Title/Abstract]) OR “post‐traumatic stress disorder*”[Title/Abstract]) OR “posttraumatic stress disorder*”[Title/Abstract]) OR “post traumatic stress disorder*”[Title/Abstract]) OR “stress response”[Title/Abstract]) OR “psycho trauma”[Title/Abstract]) OR psychotrauma[Title/Abstract]) OR “traumatic childbirth”[Title/Abstract]) AND (((((((((((((((((((“Breast Feeding”[MeSH Terms]) OR “Breast Feeding”[Title/Abstract]) OR breastfeeding[Title/Abstract]) OR Breast‐feeding[Title/Abstract]) OR breastfeed*[Title/Abstract]) OR breast‐feed*[Title/Abstract]) OR “breast feed”[Title/Abstract]) OR breastfed[Title/Abstract]) OR breast‐fed[Title/Abstract]) OR “breast fed”[Title/Abstract]) OR “breast suckling”[Title/Abstract]) OR lactation[Title/Abstract]) OR “human milk”[Title/Abstract]) OR “breastfeeding success”[Title/Abstract]) OR “breastfeeding initiation”[Title/Abstract]) OR “breastfeeding duration”[Title/Abstract]) OR “exclusive breastfeeding”[Title/Abstract]) OR Breast Feeding, Exclusive[Title/Abstract]) OR “Exclusive Breast Feeding”[Title/Abstract])

Search strategy in PsycINFO.

**Table:**

TI postpartum OR AB postpartum OR TI postnatal OR AB postnatal OR TI puerperal OR AB puerperal OR TI perinatal OR AB perinatal OR TI prenatal OR AB prenatal OR TI antenatal OR AB antenatal OR TI intrapartum OR AB intrapartum OR TI pregnancy OR AB pregnancy OR TI “pregnant women” OR AB “pregnant women” OR TI matern* OR AB matern* OR TI postpartum OR AB postpartum OR TI post‐partum OR AB post‐partum OR TI “post partum” OR AB “post partum” OR TI puerperium OR AB puerperium OR TI “Perinatal care” OR AB “Perinatal care” OR TI perinat* OR AB perinat* OR TI postnat* OR AB postnat* OR TI maternal OR AB maternal OR TI “Prenatal Care” OR AB “Prenatal Care” OR TI “Puerperal Disoders” OR AB “Puerperal Disorders” AND TI “Post‐Traumatic Stress Disorder” OR AB “Post‐Traumatic Stress Disorder” OR TI “Posttraumatic Stress Disorder” OR AB “Posttraumatic Stress Disorder” OR TI “Post Traumatic Stress Disorder” OR AB “Post Traumatic Stress Disorder” OR TI “Acute Post‐Traumatic Stress Disorder” OR AB “Acute Post‐Traumatic Stress Disorder” OR TI “Acute Post Traumatic Stress Disorder” OR AB “Acute Post Traumatic Stress Disorder” OR TI ptsd OR AB ptsd OR TI “Post‐Traumatic Neuroses” OR AB “Post‐Traumatic Neuroses” OR TI “Posttraumatic Neuroses” OR AB “Posttraumatic Neuroses” OR TI “posttraumatic neuros*” OR AB “posttraumatic neuros*” OR TI “Post‐Traumatic stress symptoms” OR AB “Post‐Traumatic stress symptoms” OR TI “Traumatic Stress Disorders” OR AB “Traumatic Stress Disorders” OR TI “Stress Disorder” OR AB “Stress Disorder” OR TI “Traumatic Stress Disorder” OR AB “Traumatic Stress Disorder” OR TI “Traumatic Stress Disorder*” OR AB “Traumatic Stress Disorder” OR TI “traumatic disorder” OR AB “traumatic disorder” OR TI “traumatic stress” OR AB “traumatic stress” OR TI “stress disorder*” OR AB “stress disorder*” OR TI post‐traumatic OR AB post‐traumatic OR TI “post traumatic” OR AB “post traumatic” OR TI posttraumatic OR AB posttraumatic OR TI posttrauma OR AB posttrauma OR TI traumatic OR AB traumatic OR TI trauma OR AB trauma OR TI “post‐traumatic stress disorder*” OR AB “post‐traumatic stress disorder*” OR TI “posttraumatic stress disorder*” OR AB “posttraumatic stress disorder*” OR TI “post traumatic stress disorder*” OR AB “post traumatic stress disorder*” OR TI “stress response” OR AB “stress response” OR TI “psycho trauma” OR AB “psycho trauma” OR TI psychotrauma OR AB psychotrauma OR TI “traumatic childbirth” OR AB “traumatic childbirth” AND TI “Breast Feeding” OR AB “Breast Feeding” OR TI breastfeeding OR AB breastfeeding OR TI Breast‐feeding OR AB Breast‐feeding OR TI breastfeed* OR AB breastfeed* OR TI breast‐feed* OR AB breast‐feed* OR TI “breast feed” OR AB “breast feed” OR TI “breast suckling” OR AB “breast suckling” OR TI lactation OR AB lactation OR TI “human milk” OR AB “human milk” OR TI “breastfeeding success” OR AB “breastfeeding success” OR TI “breastfeeding initiation” OR AB “breastfeeding initiation” OR TI “breastfeeding duration” OR AB “breastfeeding duration” OR TI “exclusive breastfeeding” OR AB “exclusive breastfeeding” OR TI “Exclusive Breast Feeding” OR AB “Exclusive Breast Feeding”

Search strategy in Scopus.

**Table:**

TITLE‐ABS(postpartum) OR TITLE‐ABS(postnatal) OR TITLE‐ABS(puerperal) OR TITLE‐ABS(perinatal) OR TITLE‐ABS(prenatal) OR TITLE‐ABS(antenatal) OR TITLE‐ABS(intrapartum) OR TITLE‐ABS(pregnancy) OR TITLE‐ABS(“pregnant women”) OR TITLE‐ABS(post‐partum) OR TITLE‐ABS(“post partum”) OR TITLE‐ABS(puerperium) OR TITLE‐ABS(“Perinatal care”) OR TITLE‐ABS(perinat*) OR TITLE‐ABS(postnat*) OR TITLE‐ABS(maternal) OR TITLE‐ABS(“Prenatal care”) OR TITLE‐ABS(“Puerperal Disorders”) AND TITLE‐ABS(“Post‐Traumatic Stress Disorder”) OR TITLE‐ABS(“Posttraumatic Stress Disorder”) OR TITLE‐ABS(“Post Traumatic Stress Disorder”) OR TITLE‐ABS(“Acute Post‐Traumatic Stress Disorder”) OR TITLE‐ABS(“Acute Post Traumatic Stress Disorder”) OR TITLE‐ABS(ptsd) OR TITLE‐ABS(“Post‐Traumatic Neuroses”) OR TITLE‐ABS(“Post Traumatic Neuroses”) OR TITLE‐ABS(“Posttraumatic Neuroses”) OR TITLE‐ABS(“posttraumatic neuros*”) OR TITLE‐ABS(“Post‐Traumatic stress symptoms”) OR TITLE‐ABS(“Traumatic Stress Disorder”) OR TITLE‐ABS(“Traumatic Stress Disorder*”) OR TITLE‐ABS(“Traumatic Disorder”) OR TITLE‐ABS(“traumatic stress”) OR TITLE‐ABS(“stress disorder*”) OR TITLE‐ABS(post‐traumatic) OR TITLE‐ABS(“post traumatic”) OR TITLE‐ABS(posttraumatic) OR TITLE‐ABS(posttrauma) OR TITLE‐ABS(traumatic) OR TITLE‐ABS(trauma) OR TITLE‐ABS(“posttraumatic stress disorder*”) OR TITLE‐ABS(“post‐traumatic stress disorder*”) OR TITLE‐ABS(“post traumatic stress disorder*”) OR TITLE‐ABS(“stress response”) OR TITLE‐ABS(“psycho trauma”) OR TITLE‐ABS(psychotrauma) OR TITLE‐ABS(“traumatic childbirth”) AND TITLE‐ABS(“Breast Feeding”) OR TITLE‐ABS(breastfeeding) OR TITLE‐ABS(Breast‐feeding) OR TITLE‐ABS(breastfeed*) OR TITLE‐ABS(breast‐feed*) OR TITLE‐ABS(“breast feed*”) OR TITLE‐ABS(breastfed) OR TITLE‐ABS(breast‐fed) OR TITLE‐ABS(“breast fed”) OR TITLE‐ABS(“breast suckling”) OR TITLE‐ABS(lactation) OR TITLE‐ABS(“human milk”) OR TITLE‐ABS(“breastfeeding success”) OR TITLE‐ABS(“breastfeeding initiation”) OR TITLE‐ABS(“breastfeeding duration”) OR TITLE‐ABS(“exclusive breastfeeding”) OR TITLE‐ABS(“exclusive breast‐feeding”) OR TITLE‐ABS(“exclusive breast feeding”)

Search strategy in Web of Science.

**Table:**

(((((((((((((((((((((((((((((((((((((TI=(postpartum)) OR AB=(postpartum)) OR TI=(postnatal)) OR AB=(postnatal)) OR TI=(puerperal)) OR AB=(puerperal)) OR TI=(perinatal)) OR AB=(perinatal)) OR TI=(prenatal)) OR AB=(prenatal)) OR TI=(antenatal)) OR AB=(antenatal)) OR TI=(intrapartum)) OR AB=(intrapartum)) OR TI=(pregnancy)) OR AB=(pregnancy)) OR TI=(“pregnant women”)) OR AB=(“pregnant women”)) OR TI=(matern*)) OR AB=(matern*)) OR TI=(post‐partum)) OR AB=(post‐partum)) OR TI=(“post partum”)) OR AB=(“post partum”)) OR TI=(puerperium)) OR AB=(puerperium)) OR TI=(“Perinatal care”)) OR AB=(“Perinatal care”)) OR TI=(perinat*)) OR AB=(perinat*)) OR TI=(postnat*)) OR AB=(postnat*)) OR TI=(maternal)) OR AB=(maternal)) OR TI=(“Prenatal care”)) OR AB=(“Prenatal care”)) OR TI=(“Puerperal Disorders”)) OR AB=(“Puerperal Disorders”) AND (((((((((((((((((((((((((((((((((((((((((((((((((((((((((((((((((((((((((TI=(“Post‐Traumatic Stress Disorder”)) OR AB=(“Post‐Traumatic Stress Disorder”)) OR TI=(“Posttraumatic Stress Disorder”)) OR AB=(“Posttraumatic Stress Disorder”)) OR TI=(“Post Traumatic Stress Disorder”)) OR AB=(“Post Traumatic Stress Disorder”)) OR TI=(“Acute Post‐Traumatic Stress Disorder”)) OR AB=(“Acute Post‐Traumatic Stress Disorder”)) OR TI=(“Acute Post Traumatic Stress Disorder”)) OR AB=(“Acute Post Traumatic Stress Disorder”)) OR TI=(ptsd)) OR AB=(ptsd)) OR TI=(“Post‐Traumatic Neuroses”)) OR AB=(“Post‐Traumatic Neuroses”)) OR TI=(“Post Traumatic Neuroses”)) OR AB=(“Post Traumatic Neuroses”)) OR TI=(“Posttraumatic Neuroses”)) OR AB=(“Posttraumatic Neuroses”)) OR TI=(“posttraumatic neuros*”)) OR AB=(“posttraumatic neuros*”)) OR TI=(“Post‐Traumatic stress symptoms”)) OR AB=(“Post‐Traumatic stress symptoms”)) OR TI=(“Traumatic Stress Disorder”)) OR AB=(“Traumatic Stress Disorder”)) OR TI=(“Traumatic Stress Disorder*”)) OR AB=(“Traumatic Stress Disorder*”)) OR TI=(“Traumatic Disorder”)) OR AB=(“Traumatic Disorder”)) OR TI=(“traumatic stress”)) OR AB=(“traumatic stress”)) OR TI=(“stress disorder”)) OR AB=(“stress disorder”)) OR TI=(post‐traumatic)) OR AB=(post‐traumatic)) OR TI=(“post traumatic”)) OR AB=(“post traumatic”)) OR TI=(posttraumatic)) OR AB=(posttraumatic)) OR TI=(posttrauma)) OR AB=(posttrauma)) OR TI=(traumatic)) OR AB=(traumatic)) OR TI=(trauma)) OR AB=(trauma)) OR TI=(“posttraumatic stress disorder*”)) OR AB=(“posttraumatic stress disorder*”)) OR TI=(“post‐traumatic stress disorder*”)) OR AB=(“post‐traumatic stress disorder*”)) OR TI=(“post traumatic stress disorder*”)) OR AB=(“post traumatic stress disorder*”)) OR TI=(“stress response”)) OR AB=(“stress response”)) OR TI=(“psycho trauma”)) OR AB=(“psycho trauma”)) OR TI=(psychotrauma)) OR AB=(psychotrauma)) OR TI=(“traumatic childbirth”)) OR AB=(“traumatic childbirth”)) AND (((((((((((((((((((((((((((((((((((TI=(“Breast Feeding”)) OR AB=(“Breast Feeding”)) OR TI=(breastfeeding)) OR AB=(breastfeeding)) OR TI=(Breast‐feeding)) OR AB=(Breast‐feeding)) OR TI=(breastfeed*)) OR AB=(breastfeed*)) OR TI=(breast‐feed*)) OR AB=(breast‐feed*)) OR TI=(“breast feed*”)) OR AB=(“breast feed*”)) OR TI=(breastfeed)) OR AB=(breastfeed)) OR TI=(breast‐fed)) OR AB=(breast‐fed)) OR TI=(“breast fed”)) OR AB=(“breast fed”)) OR TI=(“breast suckling”)) OR AB=(“breast suckling”)) OR TI=(lactation)) OR AB=(lactation)) OR TI=(“human milk”)) OR AB=(“human milk”)) OR TI=(“breastfeeding success”)) OR AB=(“breastfeeding success”)) OR TI=(“breastfeeding initiation”)) OR AB=(“breastfeeding initiation”)) OR TI=(“breastfeeding duration”)) OR AB=(“breastfeeding duration”)) OR TI=(“exclusive breastfeeding”)) OR AB=(“exclusive breastfeeding”)) OR TI=(“exclusive breast‐feeding”)) OR AB=(“exclusive breast‐feeding”)) OR TI=(“exclusive breast feeding”)) OR AB=(“exclusive breast feeding”)

Search strategy in Psycharticles.

**Table:**

TI postpartum OR AB postpartum OR TI postnatal OR AB postnatal OR TI puerperal OR AB puerperal OR TI perinatal OR AB perinatal OR TI prenatal OR AB prenatal OR TI antenatal OR AB antenatal OR TI intrapartum OR AB intrapartum OR TI pregnancy OR AB pregnancy OR TI “pregnant women” OR AB “pregnant women” OR TI matern* OR AB matern* OR TI postpartum OR AB postpartum OR TI post‐partum OR AB post‐partum OR TI “post partum” OR AB “post partum” OR TI puerperium OR AB puerperium OR TI “Perinatal care” OR AB “Perinatal care” OR TI perinat* OR AB perinat* OR TI postnat* OR AB postnat* OR TI maternal OR AB maternal OR TI “Prenatal Care” OR AB “Prenatal Care” OR TI “Puerperal Disoders” OR AB “Puerperal Disorders” AND TI “Post‐Traumatic Stress Disorder” OR AB “Post‐Traumatic Stress Disorder” OR TI “Posttraumatic Stress Disorder” OR AB “Posttraumatic Stress Disorder” OR TI “Post Traumatic Stress Disorder” OR AB “Post Traumatic Stress Disorder” OR TI “Acute Post‐Traumatic Stress Disorder” OR AB “Acute Post‐Traumatic Stress Disorder” OR TI “Acute Post Traumatic Stress Disorder” OR AB “Acute Post Traumatic Stress Disorder” OR TI ptsd OR AB ptsd OR TI “Post‐Traumatic Neuroses” OR AB “Post‐Traumatic Neuroses” OR TI “Posttraumatic Neuroses” OR AB “Posttraumatic Neuroses” OR TI “posttraumatic neuros*” OR AB “posttraumatic neuros*” OR TI “Post‐Traumatic stress symptoms” OR AB “Post‐Traumatic stress symptoms” OR TI “Traumatic Stress Disorders” OR AB “Traumatic Stress Disorders” OR TI “Stress Disorder” OR AB “Stress Disorder” OR TI “Traumatic Stress Disorder” OR AB “Traumatic Stress Disorder” OR TI “Traumatic Stress Disorder*” OR AB “Traumatic Stress Disorder” OR TI “traumatic disorder” OR AB “traumatic disorder” OR TI “traumatic stress” OR AB “traumatic stress” OR TI “stress disorder*” OR AB “stress disorder*” OR TI post‐traumatic OR AB post‐traumatic OR TI “post traumatic” OR AB “post traumatic” OR TI posttraumatic OR AB posttraumatic OR TI posttrauma OR AB posttrauma OR TI traumatic OR AB traumatic OR TI trauma OR AB trauma OR TI “post‐traumatic stress disorder*” OR AB “post‐traumatic stress disorder*” OR TI “posttraumatic stress disorder*” OR AB “posttraumatic stress disorder*” OR TI “post traumatic stress disorder*” OR AB “post traumatic stress disorder*” OR TI “stress response” OR AB “stress response” OR TI “psycho trauma” OR AB “psycho trauma” OR TI psychotrauma OR AB psychotrauma OR TI “traumatic childbirth” OR AB “traumatic childbirth” AND TI “Breast Feeding” OR AB “Breast Feeding” OR TI breastfeeding OR AB breastfeeding OR TI Breast‐feeding OR AB Breast‐feeding OR TI breastfeed* OR AB breastfeed* OR TI breast‐feed* OR AB breast‐feed* OR TI “breast feed” OR AB “breast feed” OR TI “breast suckling” OR AB “breast suckling” OR TI lactation OR AB lactation OR TI “human milk” OR AB “human milk” OR TI “breastfeeding success” OR AB “breastfeeding success” OR TI “breastfeeding initiation” OR AB “breastfeeding initiation” OR TI “breastfeeding duration” OR AB “breastfeeding duration” OR TI “exclusive breastfeeding” OR AB “exclusive breastfeeding” OR TI “Exclusive Breast Feeding” OR AB “Exclusive Breast Feeding”

## Appendix C

Inclusion and exclusion criteria based on the PICOS strategy.

**Table:**

Criteria	Inclusion criteria	Exclusion criteria
Population	Mothers whose children were born alive and healthy.	Other populations.
Key variable	Traumatic childbirth and/or PTSD/PTSS due to childbirth.	Not assessing traumatic childbirth and/or PTSD/PTSS.
Outcome	Breastfeeding outcomes.	Not assessing breastfeeding outcomes.
Study design	Observational: Prospective longitudinal or cohort study.	Other study designs.
Language	All languages.	None.
Setting	All settings.	None.

## Appendix D

Full‐text studies excluded from the systematic review.

**Table:**

Authors, year	Exclusion Criteria
Alvarenga et al., 2017	Publication type is a systematic review
Babu et al., 2022	Study design is cross‐sectional
Beck et al., 2011	Key variable not associated to outcome
Beck & Watson, 2008	Study design is qualitative
Bertholdt et al., 2020	Publication type is not a study, but a protocol
Chen et al., 2022	Key variable not associated to outcome
Cook et al., 2018	Publication type is a systematic review
Dekel et al., 2019	Study design is cross‐sectional
DeYoung & Mangum, 2021	Key variable is not traumatic childbirth and/or PTSD due to childbirth
Fajobi, 2020	Study design is cross‐sectional
Fujimori et al., 2010	Study design is qualitative
Gankanda et al., 2021	Study design is cross‐sectional
Hopkins & Hellberg, 2021	Publication type is a book chapter
Iyengar et al., 2022	Outcome of breastfeeding not assessed
Janevic et al., 2021	Study design is cross‐sectional
Kendall‐Tackett, 2022	Publication type is a book
Kendall‐Tackett, 2014	Publication type is a book
Martinez‐Vázquez et al., 2021	Study design is cross‐sectional
Mayopoulos et al., 2021	Study design is cross‐sectional
Mezzacappa, 2004	Publication type is a literature review
Mojab, 2008	Publication type is a book
Namujju et al., 2018	Study design is qualitative
Norman et al., 2022	Study design is qualitative
Orovou et al., 2020	Key variable is not traumatic childbirth and/or PTSD due to childbirth
Ples et al., 2018	Key variable is not traumatic childbirth and/or PTSD due to childbirth
Ravaldi et al., 2022	Key variable is not traumatic childbirth and/or PTSD due to childbirth
Ravaldi et al., 2020	Study design is cross‐sectional
Ravaldi et al., 2020	Study design is cross‐sectional
Rowlands & Redshaw, 2012	Key variable is not traumatic childbirth and/or PTSD due to childbirth
Srkalović et al., 2017	Key variable is not traumatic childbirth and/or PTSD due to childbirth
Van Sieleghem et al., 2022	Publication type is a systematic review
Vitale, 2021	Key variable is not traumatic childbirth and/or PTSD due to childbirth
Vukšić et al., 2022	Study design is cross‐sectional
Wang et al., 2020	Outcome of breastfeeding not assessed

## Appendix E

Quality appraisal studies using NOS scale.

**Table:**

Authors (year)	Selection				Comparability			Outcome			Total	Score
	Representativeness of the Exposed Cohort	Selection of the Non‐ exposed Cohort	Ascertainment of Exposure	Demonstration That the Outcome of Interest Was Not Present at the Start of the Study start of study	Comparability of Cohorts on the Basis of the Design or Analysis			Assessment of Outcome	Was Follow‐Up Long Enough for Outcomes to Occur	Adequacy of the Follow‐Up		
Dimitraki et al. (2016)		☆	☆	☆	☆☆			☆		☆	7	Low risk of bias
Garthus‐Niegel et al. (2018)	☆	☆	☆	☆	☆☆			☆	☆	☆	9	Low risk of bias
Halperin et al. (2015)	☆	☆	☆	☆	☆☆					☆	7	Low risk of bias
Lindau et al. (2014)	☆	☆	☆	☆	☆☆			☆	☆	☆	9	Low risk of bias
Machado et al. (2014)	☆	☆	☆	☆	☆☆			☆		☆	8	Low risk of bias
Martini et al. (2022)	☆	☆	☆	☆	☆☆			☆	☆	☆	9	Low risk of bias
Srkalović imširagić et al. (2016)	☆	☆	☆	☆	☆☆			☆		☆	8	Low risk of bias
Türkmen et al. (2020)	☆	☆	☆	☆	☆☆			☆	☆	☆	9	Low risk of bias

## Appendix F

Source of funding of the included studies in the systematic review.

**Table:**

Authors (year)	Source of funding
Dimitraki et al. (2016)	No information.
Garthus‐Niegel et al. (2018)	Norwegian Research Council Grant. Award Number: 191098.
Halperin et al. (2015)	No information.
Lindau et al. (2014)	Agency of Public Health, Lazio, and by the Italian Ministry of Health within the project of breastfeeding and atopic dermatitis (Ricerca Corrente 5.1).
Machado et al. (2014)	Fundação de Amparo à Pesquisa do Estado de Minas Gerais (FAPEMIG – Protocol APQ 00846‐11, de 2011 – Demanda Universal).
Martini et al. (2022)	Institute of Clinical Psychology and Psychotherapy, Technische Universität Dresden, Germany.
Srkalović imširagić et al. (2016)	No information.
Türkmen et al. (2020)	No funded.
